# Time to first consultation, diagnosis and treatment of TB among patients attending a referral hospital in Northwest, Ethiopia

**DOI:** 10.1186/1471-2334-14-19

**Published:** 2014-01-10

**Authors:** Solomon A Yimer, Gunnar A Bjune, Carol Holm-Hansen

**Affiliations:** 1 Department of Bacteriology and Immunology, Norwegian Institute of Public Health, Division of Infectious Disease Control, PO Box 4404, Nydalen, 0403 Oslo, Norway; 2 Institute of Health and Society, Section for International Health, Faculty of Medicine, University of Oslo, PO Box1130, Blindern, 0318 Oslo, Norway; 3 Amhara Regional State Health Bureau, Bahir Dar, PO Box 495, Bahir Dar, Ethiopia; 4 Department of Microbiology, Oslo University Hospital, PO Box 4950, Nydalen, 0424 Oslo, Norway

**Keywords:** Tuberculosis, Patients’ delay, Health systems’ delay, Tuberculosis symptoms, Ethiopia

## Abstract

**Background:**

Early detection and treatment of TB is essential for the success of TB control program performance. The aim of this study was to determine the length and analyze predictors of patients’, health systems’ and total delays among patients attending a referral hospital in Bahir Dar, Ethiopia.

**Methods:**

A cross-sectional study was conducted among newly diagnosed TB cases ≥ 15 years of age. Delay was analyzed at three levels: the periods between 1) onset of TB symptoms and first visit to medical provider, i.e. patients’ delay, 2) the first visit to a medical provider and the initiation of treatment i.e. health systems’ delay and 3) onset of TB symptoms and initiation of treatment i.e. total delay. Uni- and multi-variate logistic regression analyses were performed to investigate predictors of patients’, health systems’ and total delays.

**Results:**

The median time of patients’ delay was 21 days [(interquartile range (IQR) (7 days, 60 days)]. The median health systems’ delay was 27 days (IQR 8 days, 60 days) and the median total delay was 60 days (IQR 30 days, 121 days). Patients residing in rural areas had a three-fold increase in patients’ delay compared to those from urban areas [Adjusted Odds Ratio (AOR) 3.4; 95% (CI 1.3, 8.9)]. Extra-pulmonary TB (EPTB) cases were more likely to experience delay in seeking treatment compared to pulmonary (PTB) cases [(AOR 2.6; 95% (CI 1.3, 5.4)]. Study subjects who first visited health centres [(AOR) 5.1; 95% (CI 2.1, 12.5)], private facilities [(AOR) 3.5; 95% (CI 1.3, 9.7] and health posts [(AOR) 109; 95% (CI 12, 958], were more likely to experience an increase in health systems’ delay compared to those who visited hospitals.

**Conclusions:**

The majority of TB patients reported to medical providers within an acceptable time after the onset of symptoms. Rural residence was associated with patients’ and total delays. Providing the population with information about TB symptoms and the importance of early health seeking may be an efficient way to decrease TB transmission, morbidity and mortality. Establishing efficient TB diagnostic and treatment facilities at the periphery level is imperative to reduce diagnostic delay and expedite TB treatment.

## Background

Tuberculosis (TB) is a major public health problem in the developing world. Over eight million new cases and 1.4 million deaths occurred as a result of TB in 2012 [1]. Globally, South East Asia and sub-Saharan African countries have the highest TB burden. Ethiopia is one of the high TB burden countries in sub-Saharan Africa with an estimated incidence of 258/100,000 population. According to the 2011 Ethiopian demographic and health survey, 19.7% of the TB patients were coinfected with HIV. The adult HIV prevalence rate was estimated at 1.5% [2]. Prompt diagnosis and treatment is crucial to reduce the prevalence and incidence of TB in Ethiopia.

Delayed consultation, diagnosis and treatment of TB may lead to severe illness among patients and TB transmission in the community. A number of studies have been conducted to investigate and determine the magnitude of factors contributing to delay in TB diagnosis and treatment. A systematic review indicated that the median length of delay from the onset of symptoms to the start of treatment ranged from 60 to 90 days [3]. This delay was due to repeated visits to the same level of health care and difficulty in accessing better diagnostic and management services for TB. Diagnostic and treatment delay in TB has been studied in various regions of Ethiopia [4-8].

One of the limitations in studies addressing TB diagnostic and treatment delay is linked to the nature of study participants. Most studies only involved smear-positive cases who are the most infectious TB patients. The exclusion of extra-pulmonary (EPTB) and smear-negative TB in delay studies limits our understanding of diagnostic delay and the consequences of delay with regard to TB transmission. Both smear-negative and EPTB patients may contribute to the TB infectious pool at different levels. The relative contribution of smear-negative cases in the transmission of TB compared to smear-positive cases ranges from 20% to 22% [9,10]. In most high TB burden countries, the proportion of smear-negative cases seen in hospitals outweighs the proportion of smear-positive cases indicating that smear-negative cases may contribute significantly to TB transmission. The role of EPTB in the infectious pool is not well documented. However, it is common to see many EPTB patients with simultaneous pulmonary TB (PTB). Therefore, the possibility of transmission among EPTB-PTB cases may be high. One cannot disregard the possibility of transmission among close contacts of EPTB cases and the ultimate contribution of EPTB to the TB infectious pool.

Understanding the magnitude of and factors contributing to delay in diagnosis and treatment among all categories of TB patients is crucial in designing strategies that will improve TB control program performance. The objective of this study was to determine the length and analyze factors of patients’ , health systems’ and total delays among TB patients.

## Methods

### Setting

The study was conducted at the Felegehiwot Referal Hospital in Bahir Dar city, Amhara Region, Ethiopia. The hospital is located in the western part of the region and serves as a referral centre for patients from neighbouring zones. The majority (87.7%) of the people in the region are rural residents and live on subsistence farming. Food insecurity and deep-rooted poverty poses an enormous challenge to the population in the study area. Patients use various options to seek health care. Self-treatment, modern health providers, traditional practitioners or a combination of services may be used by patients [4]. The first level of health care in the Ethiopian health care delivery system is the health post. A health post is available in each *Kebele* (the lowest administrative unit with an average population of 5000) and is run by two salaried female health extension workers who are trained for one year in basic preventive and promotive health services. There are no diagnostic facilities for TB at health posts and patients must often travel long distances to access TB diagnostic facilities in hospitals. Various transport modalities are used to reach at the referral hospital. Some patients walk on foot, or ride horse or mule. Others may come by boat or use public transportation that may require up to seven hours before reaching the referral hospital. Most patients in rural areas walk for up to two hours to catch public transport that will take them to the hospital.

### Design and data collection

The study design was cross-sectional and the field work was conducted from January to August 2010. Newly diagnosed PTB and EPTB cases ≥ 15 years of age were enrolled consecutively in the study. A semi-structured questionnaire was used to collect data including socio-demographic and clinical factors (gender, age, education, occupation, clinical form of TB, symptoms at presentation and HIV sero-status). The data were collected by trained nurses and the questionnaire was pretested before data collection was initiated.

The national TB control guideline was followed to make diagnoses for the different categories of TB patients [11]. Smear-positive TB is diagnosed when a patient has at least two initial smear-positive sputum examinations for acid fast bacilli (AFB), when AFB is detected in one of the initial smear examinations by direct microscopy and is culture-positive, or when one initial smear-positive examination and radiographic findings are consistent with active TB as determined by a clinician. Smear-negative TB is diagnosed when a patient with symptoms suggestive of TB has at least three initial smear-negative sputum examinations for AFB and no response to a course of broad-spectrum antibiotics, when a patient has three smear-negative examinations by direct microscopy, radiological anomalies consistent with PTB and a decision by a physician to treat with a full course of anti-TB drugs, or when a patient’s diagnosis is based on culture-positive *Mycobacterium tuberculosis* despite three initial smear-negative examinations by direct microscopy. EPTB is diagnosed in patients with TB in organs other than the lungs as indicated by one culture-positive specimen from an extra-pulmonary site or histo-pathological evidence from a biopsy, or strong clinical evidence suggestive of active EPTB and the decision by a physician to start treatment with anti-TB chemotherapy.

### Data analysis

According to the literature review that we performed, there is no standard cutoff point to divide delay periods into acceptable and unacceptable delays. The acceptable length of the different delay periods in this study was thus determined by the consulting physicians and cut-off points used in previous studies [5-8]. A cut-off point of 30 days was considered acceptable for patients’ delay. A delay of more than 30 days was considered unacceptable and study participants were accordingly categorized into “delay” and “non-delay” groups. To evaluate health systems’ delay, a two week cut-off point was applied [5]; patients with delay of less than two weeks were considered “non-delay” and those with more than two weeks were considered “delayed”. To analyze predictor variables for the total delay, the sample was dichotomized into “delay” and “non-delay” taking the median total delay as a cut-off point. Delay beyond the median total delay value was considered unacceptable.

Data analysis was performed using Statistical Package for the Social Sciences (SPSS) version 20 (Chicago, Illinois). Data were presented as the number of cases and proportions. Means and medians with interquartile range (IQR) were calculated. Uni- and multi-variate logistic regression analysis was used to evaluate the factors associated with the different delay periods. Uni-variate analysis was performed first on each variable to calculate crude odds ratios. Epidemiological and clinical significance of the variables was considered for inclusion in the regression model. The goodness of fit of the multiple logistic regression model was assessed using the Hosmer-Lemeshow test. A two-sided p value of less than 0.05 was considered statistically significant.

### Definition of variables

Patients’ delay: the period from the start of TB symptoms until first presentation to a medical provider.

Health systems’ delay: the period from first presentation to a medical provider until first diagnosis.

Total delay: the period from start of TB symptoms until start of treatment.

Medical providers: modern health care facilities such as health posts, clinics, health centres or hospitals owned by the government or the private sector.

Ethical clearance was secured from the Regional Committee for Medical Research Ethics in Eastern Norway (REK Øst) and the Ethiopian Science and Technology Agency in Addis Ababa, Ethiopia. All study participants provided informed consent prior to study start.

## Results

### Characteristics of the study population

A total of 201 TB patients were included in the study; 124 (61.7%) men and 77 (38.3%) women with a mean age of 31 years. The mean age for EPTB cases (27 years) was lower than PTB cases (33 years) (p < 0.002) (Table 1).

**Table 1:** Socio-demographic and clinical characteristics of the study participants attending Felegehiwot Referral Hospital in Bahir Dar, Ethiopia

**Variable**	**N (%)**	**P value**
Sex		
Male	124 (61,7)	
Female	77 (38.3)	0.001
Age		
15-34	133 (66.2)	
35-54	53 (26.4)	0.000
>55	14 (7.0)	
Marriage		
Married	113 (56.2)	
Single	66 (32.8)	0.000
Widowed	7 (3.5)	
Divorced	15 (7.5)	
Residence		
Urban	86 (42.8)	
Rural	115 (57.2)	0.41
Education		
Below 8^th^ grade	135 (67.2)	
Secondary	32 (15.9)	
College and above	34 (16.9)	0.000
Employment		
Peasants	87 (43.3)	
Civil servant	20 (10.0)	
Others	94 (46.8)	0.000
HIV sero-status		
Positive	42 (20.9)	
Negative	128 (63.7)	0.000
Not known	31 (15.4)	
Patient category		
Smear-positive	42 (20.9)	
Smear-negative	78 (38.8)	0.000
Extra pulmonary	81 (40.3)	
Income		
Less than 300 birr^§^	137 (68.2)	
Above 300 birr	64 (31.8)	0.000
No people		
1-2	49 (24.4)	
3-4	67 (33.3)	0.008
5 and above	85 (42.3)	
No rooms		
1	166 (82.6)	
2	22 (10.9)	0.000
3 and above	13 (6.5)	

^§^300 Birr = USD 16.

### TB Symptoms

Patients experienced various TB symptoms (Table 2). Significant differences in TB symptoms were observed between PTB and EPTB patients. Cough (73.3% PTB, 2.5% EPTB, p = 0.000), chest pain (90.8% PTB, 6.2% EPTB, p = 0.000), and loss of appetite (95.8% PTB, 84.0% EPTB, p = 0.004) were more common among PTB than EPTB patients. Rheumatic pain (5.8% PTB, 58.8% EPTB, p = 0.000) was more frequent among EPTB than PTB patients.

**Table 2:** TB symptoms experienced by TB patients attending Felegehiwot Referral Hospital in Bahir Dar, Ethiopia

**Smear-positive**	**N (%)**	**Smear-negative**	**N (%)**	**EPTB**	**N (%)**
Cough	42 (100)	Fever	77 (98.7)	Night sweating	80 (98.8)
Night sweating	41 (97.6)	Night sweating	76 (97.4)	Fever	74 (91.4)
Loss of appetite	41 (97.6)	Loss of appetite	74 (94.9)	Weight loss	71 (87.7)
Weight loss	40 (95.2)	Weight loss	72 (92.3)	Loss of appetite	68 (84.0)
Fever	40 (95.2)	Chest pain	70 (89.7)	Malaise	60 (74.1)
Chest pain	39 (92.5)	Cough	69 (88.5)	Rheumatic pain	47 (58.0)
Dyspnea	38 (90.5)	Dyspnea	61 (78.2)	Chest pain	5 (6.2)
Malaise	33 (78.6)	Malaise	50 (64.1)	Cough	3 (3.7)
Haemoptysis	18 (42.9)	Haemoptysis	15 (19.2)		
Rheumatic pain	1 (2.4)	Rheumatic pain	6 (7.7)		

### Length of delay

The median time of patients’ delay was 21 days (IQR 7 days, 60 days). The median health systems’ delay was 27 days (IQR 8 days, 60 days), and the median total delay was 60 days (IQR 30 days, 121 days). One hundred thirty eight (68.7%) of the study participants reported to medical providers less than one month after the start of symptoms. For 132 (68.7%) of the participants, the period from first visit to a medical provider until diagnosis took more than two weeks. The total delay for 92 (47.8%) of the cases exceeded two months.

### Predictors of delay

Patients residing in rural areas had a three-fold increase in patients’ delay compared to patients from urban areas [Adjusted Odds Ratio (AOR) 3.4; 95% (CI 1.3, 9.0)]. Patients with EPTB were more likely to experience delay in seeking treatment compared to PTB cases [AOR 2.6; 95% (CI 1.3, 5.4)] (Table 3). HIV-positive patients were less likely to have increased health systems’ delay compared to HIV-negative patients [AOR 0.2; 95% CI (0.1, 0.5)]. Patients who first visited health centres [AOR 5.1; 95% (CI 2.1, 12.5)], private facilities [AOR 3.5; 95% (CI 1.3, 9.7)] and health posts [AOR 109; 95% (CI 12, 950)] had longer health systems’ delay compared to those who visited hospitals (Table 4). Patients with EPTB [AOR 3.2; 95% (CI 1.5, 7.0)] and rural residents [AOR 2.8; 95% CI (1.1, 7.3)] had increased risk of total delay compared to PTB cases and urban dwellers, respectively. Patients above 55 years of age had a higher risk of total delay compared to patients 15–34 years of age [AOR 1.9; 95% (CI 3.3, 10.9)] (Table 5).

**Table 3:** Associations of patient delay with socio-demographic and clinical factors among TB patients attending Felegehiwot Referral Hospital in Bahir Dar, Ethiopia

**Variable**	**N (%)**	**Delayed (%)**	**Crude odds ratio 95% CI**	**Adjusted odds ratio 95% CI**
Sex				
Male	124 (61,7)	40 (32.3)	1	1
Female	77 (38.3)	23 (29.9)	0.89 (0.5, 1.7)	0.5 (0.3, 1.1)
Age				
15-34	133 (66.2)	39 (29.3)	1	1
35-54	53 (26.4)	17 (32.1)	1.1 (0.6, 2.3)	0.8 (0.3, 1.9)
>55	14 (7.0)	7 (50.0)	2.4 (0.8, 7.3)	2.2 (1.0, 8.5)
Marriage				
Married	113 (56.2)	36 (31.9)	1	1
Single	66 (32.8)	17 (25.8)	0.7 (0.3, 1.5)	0.7 (0.3, 1.7)
Widowed	7 (3.5)	3 (42.9)	1.60 (0.3, 7.6)	3.9 (0.6, 26.2)
Divorced	15 (7.5)	7 (46.7)	1.87 (0.6, 5.6)	2.6 (0.8, 8.9)
Residence				
Urban	86 (42.8)	16 (18.6)	1	1
Rural	115 (57.2)	47 (40.9)	3.02 (1.6, 5.8)*	3.4 (1.3, 8.9)*
Education				
Below 8^th^ grade	135 (67.2)	49 (36.3)	1	1
Secondary	32 (15.9)	7 (21.9)	2.20 (0.9, 5.4)	0.6 (0.2, 2.1)
College and above	34 (16.9)	7 (20.6)	1.08 (0.3, 3.5)	1.1 (0.3, 3.4)
Employment				
Peasants	87 (43.3)	36 (41.4)	1	1
Civil servant	20 (10.0)	2 (10.0)	0.16 (0.0, 0.7)*	0.3 (0.0, 2.4)
Others	94 (46.8)	25 (26.6)	0.52 (0.3, 1.0)	1.3 (0.4, 3.8)
HIV serostatus				
Negative	128 (63.7)	42 (32.8)	1	1
positive	42 (20.9)	12 (28.6)	0.8 (0.4, 1.8)	1.7 (0.6, 4.4)
Not known	31 (15.4)	9 (29.0)	1.8 (0.5, 1.9)	0.6 (0.2, 1.7)
Type of TB				
Pulmonary	120 (59.7)	31 (25.8)	1	1
Extrapulmonary	81 (40.3)	312 (39.5)	1.86 (1.0, 3.4)*	2.6 (1.3, 5.4)*
Income				
Less than 300 birr^§^	137 (68.2)	47 (34.3)	1	1
Above 300 birr	64 (31.8)	16 (25.6)	0.6 (0.3, 1.2)	0.9 ( 0.4, 2.2)

^§^300Birr = USD 16; *significantly associated.

**Table 4:** Associations of health systems’ delay with sociodemographic and clinical factors among TB patients attending Felegehiwot Referral Hospital in Bahir Dar, Ethiopia

**Variable**	**N (%)**	**Delayed (%)**	**Crude odds ratio 95% CI**	**Adjusted odds ratio 95% CI**
Sex				
Male	124 (61,7)	84 (67.7)	1	1
Female	77 (38.3)	48 (62.3)	0.8 (0.4, 1.4)	0.8 (0.3, 1.7)
Age				
15-34	133 (66.2)	87 (65.4)	1	1
35-54	53 (26.4)	34 (64.2)	0.9 (0.5, 1.8)	0.9 (0.4, 2.7)
>55	14 (7.0)	10 (71.4)	1.3 (0.4, 4.5)	0.5 (0.1, 2.7)
Marriage				
Married	113 (56.2)	76 (67.3)	1	1
Single	66 (32.8)	44 (66.7)	0.9 (0.5, 1.9)	0.7 (0.2, 1.8)
Widowed	7 (3.5)	3 (42.9)	0.4 (0.1, 1.7)	0.9 (0.1, 7.7)
Divorced	15 (7.5)	9 (60.0)	2.1 (0.2, 2.2)	1.0 (0.2, 4.3)
Residence				
Urban	86 (42.8)	43 (50.0)	1	1
Rural	115 (57.2)	89 (77.4)	3.4 (1.9, 6.3)*	2.2 (0.8, 6.0)
Education				
Below 8^th^ grade	135 (67.2)	94 (69.6)	1	1
Secondary	32 (15.9)	19 (59.4)	0.6 (0.3, 1.4)	1.8 (0.5, 6.4)
College and above	34 (16.9)	19 (55.9)	0.6 (0.3, 1.2)	0.8 (0.2, 2.6)
Employment				
Peasants	87 (43.3)	66 (75.9)	1	1
Civil servant	20 (10.0)	10 (50.0)	0.3 (0.1, 0.9)*	1.4 (0.2, 8.0)
Others	94 (46.8)	56 (59.6)	0.5 (0.3, 0.8)*	2.2 (0.6, 7.7)
Income				
Less than 300 birr^§^	137 (68.2)	77 (64.2)	1	1
Above 300 birr	64 (31.8)	55 (67.9)	0.7 (0.4, 1.4)	0.9 (0.4, 2.2)
HIV serostatus				
Negative	128 (63.7)	94 (73.4)	1	1
Positive	42 (20.9)	15 (35.7)	0.2 (0.1, 0.4)*	0.2 (0.1, 0.5)*
Not known	31 (15.4)	23 (74.2)	1.0 (0.4, 14.4)	0.5 (0.1, 1.4)
Type of TB				
Pulmonary	120 (59.7)	93 (67.9)	1	1
Extrapulmonary	81 (40.3)	39 (60.9)	1.2 (0.7, 2.2)	0.8 (0.3, 1.7)
FHP visited				
Hospital	55 (27.4)	18 (32.7)	1	1
Health centre	65 (32.3)	47 (72.3)	5.4 (2.5, 11.7)*	5.1 (2.1, 12.5)*
Private	36 (17.9)	23 (63.9)	3.6 (1.5, 8.7)*	3.5 (1.3, 9.7)*
Health post	45 (22.4)	44 (97.8)	90.4 (11.5, 710.0)*	109 (12, 958)*

^§^300Birr = USD 16.

*significantly associated.

**Table 5:** Associations of total delay with socio-demographic and clinical factors among TB patients attending Felegehiwot Referral Hospital in Bahir Dar, Ethiopia

**Variable**	**N (%)**	**Delayed (%)**	**Crude Odds ratio 95% CI**	**Adjusted odds ratio 95% CI**
Sex				
Male	124 (61,7)	54 (43.5)	1	1
Female	77 (38.3)	38 (49.4)	1.3 (0,7, 2.3)	0.7 (0.3, 1.5)
Age				
15-34	133 (66.2)	58 (43.6)	1	1
35-54	53 (26.4)	23 (43.4)	0.9 (0.5, 1.9)	0.2 (0.2, 1.3)
>55	14 (7.0)	10 (71.4)	3.2 (0.9, 10.8) *	1.9 (3.3, 10.9) *
Marriage				
Married	113 (56.2)	52 (46.0)	1	1
Single	66 (32.8)	28 (42.4)	1.6 (0.5, 1.6)	0.4 (0.2, 1.1)
Widowed	7 (3.5)	4 (57.1)	1.3 (0.3, 7.3)	15.5 (1.1, 218)
Divorced	15 (7.5)	8 (53.3)	1.3 (0.5, 3.9)	4.9 (0.9, 26.5)
Residence				
Urban	86 (42.8)	22 (25.6)	1	1
Rural	115 (57.2)	70 (60.9)	4.5 (2.6, 8.4)*	2.8 (1.1, 7.3)*
Education				
Below 8^th^ grade	135 (67.2)	73 (54.1)	1	1
Secondary	32 (15.9)	12 (37.5)	0.5 (0.2, 1.1)	0.5 (0.2, 1.4)
College and above	34 (16.9)	7 (20.6)	0.2 (0.1, 0.5)*	0.5 (0.2, 1.5)
Employment				
Peasants	87 (43.3)	53 (60.9)	1	1
Civil servant	20 (10.0)	4 (20.0)	0.2 (0.1, 0.5)*	0.3 (0.1, 1.7)
Others	94 (46.8)	35 (37.2)	0.4 (0.2, 0.7)*	1.5 (0.4, 5.0)
Income				
Less than 300 birr^§^	137 (68.2)	68 (49.6)	1	1
Above 300 birr	64 (31.8)	24 (37.5)	0.6 (0.3, 1.1)	1.1 (0.5, 2.5)
HIV serostatus				
Negative	128 (63.7)	61 (47.7)	1	1
Positive	42 (20.9)	13 (31.0)	0.4 (0.2, 0.8)*	0.7 (0.3, 1.8)
Not known	31 (15.4)	18 (58.1)	0.8 (0.4, 1.9)	0.6 (0.2, 1.7)
Type of TB				
Pulmonary	120 (59.7)	44 (36.7)	1	1
Extra-pulmonary	81 (40.3)	48 (59.3)	2.5 (1.4, 4.5)	3.2 (1.5, 7.0)*
FHP visited				
Hospital	55 (27.4)	15 (8.2)	1	1
Health centre	65 (32.3)	30 (46.2)	1.5 (0.7, 3.2)*	1.3 (0.5, 3.1)
Private	36 (17.9)	16 (45.8)	0.9 (0.4, 2.2)*	1.1 (0.4, 3.2)
Health post	45 (22.4)	32 (71.1)	3.8 (1.5, 9.7)*	2.9 (0.9, 9.1)

^§^300 Birr = USD 16.

*significantly associated.

## Discussion

While the majority of the participants in this study reported to medical providers within 30 days following the onset of symptoms, diagnosis and the start of chemotherapy was delayed. This is in accordance with the results of some previous studies [5,6,12-14] but in contrast to other studies that reported longer patients’ and shorter health systems’ delays [7,15,16].

Our findings indicate that the major problem in TB diagnosis is the amount of time used by the health system to respond to patients’ needs. The health system needs to be improved so that patients receive efficient health care when they report to medical providers. Regular refresher training courses for health extension workers and other health care providers about the importance of recognizing symptoms indicative of TB will help to reduce health system delays. A rapid serological screening test for active TB that is suitable for use at the health post will be advantageous. Patients with positive rapid test results will be referred on the same day to the next level of health care for confirmatory testing and treatment. Establishing a new molecular method for the detection of *Mycobacterium tuberculosis*, GeneXpert, at selected health centres and referral hospitals will also serve to expedite diagnosis and treatment of TB.

The observed median total delay is lower than the findings of a former study conducted in the study area [5]. This may be related to the improved access to TB care provided through the current health extension program in Ethiopia. We have previously suggested the application of “treatment delay” as an indicator of the quality of TB control program performance at the local level [17]. In this regard, the lower median total delay observed in the current study may demonstrate an improved performance of the local TB control program in the study area.

Patients with EPTB experienced long patients’ and total delays compared to PTB cases. This observation is similar with the results of other studies [3,18-22]. Most of the EPTB patients in our study were cervical TB lymphadenitis cases. In rural Ethiopia, a patient with a chronic wound, lump or mass in the neck is perceived to have *nekersa*, and it is a common practice for such patients to visit traditional healers or herbalists before reporting to medical providers [23]*.* In a recent investigation conducted among private practitioners in the study region, a considerable proportion of study participants with EPTB visited the practitioners with complications following treatment with herbal medications [24]. We have also previously interviewed cervical TB lymphadenitis cases from the study area and observed that some patients discontinued their anti-TB medication and started herbal medicine (unpublished data). It is thus likely that many of the EPTB cases might have visited traditional healers or herbalists and thereby experienced delay in reporting to medical providers. Discontinued TB regimens among EPTB patients may lead to drug resistance. Public awareness about the importance of early health seeking and the nature of EPTB among the population needs to be improved.

Most of the peripheral health institutions in the study region do not have facilities for diagnosing EPTB. The observed increased total delay among EPTB cases may reflect the lack of available diagnostic facilities. EPTB patients initially receive broad spectrum antibiotics and are referred to major towns for histopathology and other TB examinations. This leads to a long delay before starting standard TB treatment.

Rural residence affected patients’ reporting, diagnoses and treatment start. A similar finding was observed in other countries [25,26]. Targeted health education and expanding diagnostic and treatment facilities for TB in rural areas is warranted. Further qualitative research is also needed to investigate the possible reasons for delay among rural residents.

Patients above 55 years of age had an increased risk of total delay that may be related to several factors. Older patients are generally dependent on others and thus it may be difficult for them to seek health care when needed. TB disease may not exhibit the main symptoms indicative of TB (i.e. cough, haemoptysis, fever, night sweats and weight loss) in older patients. The clinical presentation of TB in older people may be indicated by changes in daily activity, anorexia, chronic fatigue, cognitive impairment or unexplained low-grade fever [27]. TB diagnosis among this age group may thus be delayed because of a low index of suspicion by health care providers. Health workers must consider nonspecific signs and symptoms persisting from weeks to months that may indicate TB among older patients.

Cough, chest pain and loss of appetite were more pronounced among PTB cases than EPTB patients. This was not surprising as the lungs are primarily involved in PTB cases. More EPTB patients suffered from rheumatic pain. Further studies should investigate symptoms that are specifically associated with ETPB.

The proportion of HIV-positive individuals among those tested was 24.7% and is higher than the national estimate of 8% for 2011 [1]. Patients with HIV-infection experienced no delay in TB diagnosis. It is possible that HIV-positive TB patients in our study suffered more serious symptoms indicative of TB and were thus immediately referred by the health care worker to a clinic or hospital for testing and treatment [28]. It is also possible that health workers have a high index of suspicion for TB when examining persons with HIV-infection. In contrast, some studies reported HIV as a risk factor for delay in TB diagnosis due to nonspecific test results and atypical clinical features [29,30]. No association between HIV serostatus and delayed TB diagnosis was reported in other studies [31,32].

The World Health Organization recommends provider-initiated HIV counselling and testing (PICT) service to all TB patients [33]. We found a considerable number of TB patients who had not been tested for HIV. It is important for TB patients to know their HIV status in order to ensure that they will receive appropriate treatment, care and support services for HIV [34]. This finding demonstrates that PICT is not optimally implemented in the study site and should be improved.

A very high proportion of patients had a family residence of only one room. The majority of families constituted more than five individuals per household. TB is a disease of poverty that commonly occurs in crowded living conditions.

The study has several limitations. Firstly, patients were recruited from a single governmental hospital. Similar patients who may have visited other hospitals, stayed at home or reported to private health providers were not included in this study. Thus it is difficult to generalize our results to all TB patients in the study region. Secondly, patients’ memory of symptoms may be subject to recall bias. We used the local calendar listing the main religious and national days to estimate the date of onset of symptoms to minimize the possibility of recall bias. Despite these concerns, we believe that the study provides baseline information about delay in diagnosis and treatment among all categories of TB patients in a referral hospital setting.

## Conclusions

In conclusion, the study demonstrated that the majority of TB patients sought health care within an acceptable time following the start of symptoms. Health systems’ and total delays were unacceptably high. Rural residence was associated with a longer delay in presenting to medical providers. Patients with EPTB and those who first visited health posts, health centres and private facilities were more likely to experience a longer health systems’ delay. While rural residence and crowded living conditions cannot be changed in the near future, a well-designed information education, communication/behavioural change communication (IEC/BCC) strategy for TB might improve the TB control program. Health education directed towards people in rural areas that explains how to suspect and recognize TB symptoms and the importance of early health seeking is needed. Regular refresher training courses for health extension workers and other health care providers that address the importance of high index of suspicion for TB may help to reduce patient and health systems factors responsible for delay. Further decentralization of TB diagnostic and treatment facilities to the periphery is highly recommended to reduce transmission and the size of the TB infectious pool in the community.

## Competing interests

The authors declare that they have no competing interests.

## Authors’ contributions

SAY, CHH and GAB designed the study. SAY conducted the data collection. SAY performed the data analysis. SAY drafted the manuscript. The three authors edited and approved the final manuscript.

## Pre-publication history

The pre-publication history for this paper can be accessed here:

http://www.biomedcentral.com/1471-2334/14/19/prepub
